# Implications of the Introduction of Cholera to Haiti

**DOI:** 10.3201/eid1707.110625

**Published:** 2011-07

**Authors:** Scott F. Dowell, Christopher R. Braden

**Affiliations:** Author affiliation: Centers for Disease Control and Prevention, Atlanta, Georgia, USA

**Keywords:** waterborne infections, cholera, Vibrio cholerae, public health, Haiti, expedited, commentary, bacteria

With more than 250,000 cases and 4,000 deaths in the first 6 months, the cholera epidemic in Haiti has been one of the most explosive and deadly in recent history. It is also one of the best documented, with detailed surveillance information available from the beginning of the epidemic, which allowed its spread to all parts of the country to be traced. Piarroux et al. make good use of this information, along with their own careful field investigations, to trace the epidemic to its beginning and propose an explanation for its origins (1).

Multiple lines of evidence indicate that *Vibrio cholerae* was newly introduced into Haiti. Cholera had not been documented in Haiti for at least several generations. Although there was a reference to a small number of “cholera” cases during the American occupation in 1928 (2), there was no culture confirmation, and the likelihood is these represented cases of severe diarrhea caused by other pathogens. In the current situation, 14 *V. cholerae* isolates from the Artibonite Department early in the epidemic were indistinguishable by multiple phenotypic and molecular characterization methods, which indicated the infections were due to a single clone of *V. cholerae* introduced into Haiti in 1 event (3). Piarroux et al. present strong evidence that the earliest cases of cholera occurred in the upper Artibonite valley, near the town of Mirebalais (1). Within days of the Mirebalais cases, hundreds of additional cases were reported in the lower Artibonite valley, more than 50 km away, and over the next 2 days in neighboring departments outside the Artibonite valley. This rapid spread is characteristic of cholera transmission in a mobile, immunologically naive population with widespread exposure to sewage-contaminated drinking water. The mobility of the population ensured transmission of the pathogen to all 10 departments of Haiti within weeks, and the uniformly poor water and sanitation infrastructure ensured that where the pathogen was introduced local transmission would follow. Indeed, residents of the camps for internally displaced persons, where chlorinated water and sanitation was in many instances provided by outside organizations, experienced relatively low numbers of cholera infections (1).

Piarroux et al. provide circumstantial evidence that fecal contamination of a local stream draining into the Artibonite River initiated the epidemic, that further spread then occurred to more heavily populated towns downstream in the river valley, and that a foreign peacekeeping battalion may have been the source of *V. cholerae* introduction into Haiti. The origin of cholera in Haiti is the subject of study by an independent panel appointed by the Secretary General of the United Nations. Certainly the spread within days to remote departments outside the valley indicates an important role for travel of infected persons along land routes in the subsequent if not the initial spread. However it occurred, there is little doubt that the organism was introduced to Haiti by a traveler from abroad, and this fact raises important public health considerations.

Introduction into an immunologically naive population was necessary but not sufficient for such explosive epidemic spread. During the course of this epidemic, there were multiple introductions into camps for internally displaced persons in Haiti, with limited spread; into communities in the Dominican Republic, resulting in local but not widespread transmission; and into Florida and other states in the United States, with no secondary spread and no epidemics (4). These populations were also immunologically naive to cholera but were protected from exposure by physical and chemical barriers—the infrastructure for water treatment and distribution and for the collection and treatment of fecal waste—that effectively prevent contamination of food and drinking water by enteric pathogens.

International travelers, including those going to serve vulnerable populations, are potential carriers of epidemic-prone disease. These travelers and their service organizations should take appropriate precautions (such as vaccination and chemoprophylaxis) to protect themselves and to forestall introducing such pathogens to local populations (5).

After travelers’ arrival in a country, the spread of cholera and other enteric infections should be prevented by ensuring adequate sanitation such that pathogens excreted by either symptomatic or asymptomatic persons cannot be introduced into local food or water supplies. Prompt diagnosis and treatment of acute illnesses that may arise will also limit the opportunities for further spread. Realistically, however, it is impossible to completely guard against the introduction of infectious diseases, including cholera, into new populations and places, given the mobility of vast numbers of persons, animals, and products around the globe.

For Haiti, the future course of the cholera epidemic is difficult to predict, especially given the chronic degradation of water and sanitation infrastructure over many years and the acute disruption from the earthquake in Haiti in January 2010 (6). Improving water and sanitation infrastructure is clearly the most effective and lasting approach to prevent the spread of cholera in countries where it is endemic as well as in those that are currently cholera-free. In the United States, Europe, and worldwide, disinfection of municipal water supplies and improvements in sanitation have dramatically reduced the incidence of cholera, typhoid, and overall mortality (7). In Haiti, the short-term public health response has included real-time surveillance, laboratory confirmation of infections, training of health workers on case management, and public education for basic cholera prevention. Enhanced access to medical services were scaled up quickly among many partner organizations by using the existing clinic infrastructure supported by the US President’s Emergency Plan for AIDS Relief. These efforts helped to reduce mortality to <1% within 4 months and bring the epidemic temporarily under control. Even as we continue to learn more about the intercontinental spread of this ancient human scourge, we are reminded of the continued effectiveness of traditional public health control measures.
